# Primary care endorsement letter and a patient leaflet to improve participation in colorectal cancer screening: results of a factorial randomised trial

**DOI:** 10.1038/bjc.2011.255

**Published:** 2011-08-09

**Authors:** P Hewitson, A M Ward, C Heneghan, S P Halloran, D Mant

**Affiliations:** 1 Department of Primary Health Care, University of Oxford, Old Road, Headington, Oxford OX3 7LF, UK; 2 Postgraduate Medical School, University of Surrey, Surrey, GU2 7WG, UK

**Keywords:** colorectal cancer, cancer screening, primary care, patient information

## Abstract

**Background:**

The trial aimed to investigate whether a general practitioner's (GP) letter encouraging participation and a more explicit leaflet explaining how to complete faecal occult blood test (FOBT) included with the England Bowel Cancer Screening Programme invitation materials would improve uptake.

**Methods:**

A randomised controlled 2 × 2 factorial trial was conducted in the south of England. Overall, 1288 patients registered with 20 GPs invited for screening in October 2009 participated in the trial. Participants were randomised to either a GP's endorsement letter and/or an enhanced information leaflet with their FOBT kit. The primary outcome was verified with return of the test kit within 20 weeks.

**Results:**

Both the GP's endorsement letter and the enhanced procedural leaflet, each increased participation by ∼6% – the GP's letter by 5.8% (95% CI: 4.1–7.8%) and the leaflet by 6.0% (95% CI: 4.3–8.1%). On the basis of the intention-to-treat analysis, the random effects logistic regression model confirmed that there was no important interaction between the two interventions, and estimated an adjusted rate ratio of 1.11 (*P*=0.038) for the GP's letter and 1.12 (*P*=0.029) for the leaflet. In the absence of an interaction, an additive effect for receiving both the GP's letter and leaflet (11.8%, 95% CI: 8.5–16%) was confirmed. The per-protocol analysis indicated that the insertion of an electronic GP's signature on the endorsement letter was associated with increased participation (*P*=0.039).

**Conclusion:**

Including both an endorsement letter from each patient's GP and a more explicit procedural leaflet could increase participation in the English Bowel Cancer Screening Programme by ∼10%, a relative improvement of 20% on current performance.

Colorectal cancer (CRC) is the second leading cause of cancer deaths in the United Kingdom (CRUK Cancerstats), and survival rates are lower than in other European countries (Berrino *et al*, 2007). Only 13% of UK CRC cases are diagnosed at the earliest stage of the disease (NCIN, 2009). As screening using the faecal occult blood test (FOBT) can increase early-stage diagnosis, significantly reducing bowel cancer mortality (Hewitson *et al*, 2007), a national screening programme was introduced in spring 2006 and it currently covers all areas of England.

The England Bowel Cancer Screening Programme invites men and women aged 60–69 years (increased to 60–74 years from April 2010) to participate in faecal occult blood screening in every 2 years. Eligible people receive an invitation letter and information booklet by post. A week later (unless they decline participation during that week), they receive the test kit, which includes brief instructions and a freepost envelope for return of the kit. Currently, around half (49–52%) of the eligible population return their test kits (Weller *et al*, 2006; von Wagner *et al*, 2009). Although these participation rates are comparable with other European screening programmes (Denis *et al*, 2007), increasing informed participation may result in a larger reduction in mortality (Parkin *et al*, 2008) and previous research has consistently indicated that the involvement of general practitioners (GPs) may be beneficial (Brawarsky *et al*, 2004; Subramanian *et al*, 2004; Senore *et al*, 2010). At present, GPs in the United Kingdom are not directly involved in bowel cancer screening – they only receive a copy of the results letters sent to their patients.

The simplest method of involving primary care in the screening process is for the invitation to participate to include a personal letter from the GP (at present the invitation comes from a Screening Hub not previously known to the patient). Although increasing participation can be achieved by repeated invitations to non-responders (Steele *et al*, 2010), evidence from other countries suggests that an endorsement letter from the patient's GP can significantly improve participation (Cole *et al*, 2007; Senore *et al*, 2010; Zajac *et al*, 2010). However, the GP's endorsement letter seems to affect only a proportion of the invited population. The potential improvement in participation through inclusion of a GP's signature on the endorsement letter was demonstrated by Cole *et al* (2002), although other studies have not shown a significant increase in participation using this method (Segnan *et al*, 2005; Ling *et al*, 2009).

Other patient-specific factors such as poor knowledge of the benefits of bowel cancer screening, concerns about performing the FOBT and perceived self-efficacy for completing the bowel cancer screening procedure can also contribute to poor participation (Seeff *et al*, 2004; Subramanian *et al*, 2004; Weller *et al*, 2006). Another potential difficulty concerns the effect of limited health literacy for the screening participation. People with limited health literacy have lower participation rates and report more barriers to complete FOBT than people with adequate health literacy (Dolan *et al*, 2004; Peterson *et al*, 2007). Addressing these difficulties, several primary care-based studies suggest providing patients with detailed instructions on the collection, storage and return of test kits, can increase compliance (Miller *et al*, 2005; Stokamer *et al*, 2005). Coupling this strategy of providing potential screening participants with more detailed instructions based on social cognitive approaches to alleviate misconceptions about screening (Bandura, 2004) and the presentation of risk information in a more effective manner (Rothman and Kiviniemi, 1999; Rothman *et al*, 2006) may positively effect FOBT participation.

We, therefore, report here the results of a factorial trial to assess the impact on participation in the English Screening Programme including the test kit: (1) a letter from the patient's GP, recommending participation in the programme and (2) an information leaflet explaining more explicitly how to undertake FOBT.

## Materials and methods

A 2 × 2 factorial randomised controlled trial design was adopted to assess the effectiveness of the two interventions – a GP's letter and/or a leaflet giving more explicit information on how to carry out and return the FOBT. Factorial trials have a number of advantages over the standard parallel group design. First, they enable efficient simultaneous investigation of two interventions by including all participants in both the analyses and second, the factorial trial can consider both the separate effects of the intervention and the benefits of receiving both interventions together (McAlister *et al*, 2003; Montgomery *et al*, 2003). Finally, the design can reduce the total number of participants required to assess the multiple interventions aimed at achieving the same outcome (Montgomery *et al*, 2003; Gurusamy *et al*, 2011).

### Interventions

The GP's endorsement letter was a personally addressed letter from each patient's GP, which: (1) recommended that the patient complete the test; (2), offered support if the patient had any questions about screening; (3) emphasised the importance of being aware of bowel cancer symptoms. We included several key messages in the letter, based on the views of the pilot participants, influential statements reported in previous UK research (Woodrow *et al*, 2008) and phrased using a ‘gain-frame’ approach, identified as important when targeting detection behaviours (Rothman *et al*, 2006). The key messages included in the endorsement letter indicated the risk of developing bowel cancer was highest in the patients’ age group; there are often no symptoms associated with early bowel cancer and screening can detect bowel cancer at an early stage. In total, 8 of the 20 practices involved provided electronic GP's signatures; the others were sent ‘on behalf of the practice’.

The enhanced procedural leaflet was extensively piloted and revised with 109 people previously invited to bowel cancer screening (see Appendices), and was modified with advice from an expert steering group involved in the trial. Pilot respondents indicated that the leaflet was easy to read (95%), sufficiently detailed (98%), and included ‘very useful’ information for collecting samples (76%), storing samples (75%) and for the decision to participate or not in screening (81%). The leaflet addressed potential barriers to screening identified from the pilot study and previous research (Subramanian *et al*, 2004; Weller *et al*, 2006; Woodrow *et al*, 2008). On the basis of social cognitive theory (Bandura, 2004) and effective methods for improving risk communication (Rothman and Kiviniemi, 1999), the leaflet included an educational or knowledge-building component (reinforcing messages regarding the effectiveness and rationale for screening) and motivational components designed to improve self-efficacy (advice on how to collect samples, concerns about time required, and what people with loose or irregular bowel motions should do).

### Recruitment and participants

Approximately 25 000 people from more than 920 GP practices were invited for bowel cancer screening during October 2009 by the Southern Programme Hub. Following a consultation with the National Bowel Cancer Screening Programme, 20 of 88 eligible GPs, in southern England, whose patients were scheduled to receive a screening invitation in the month of October 2009, agreed to participate. We sent patients from these practices a preliminary letter in August 2009 (6 weeks before their scheduled invitation date in October). This letter informed them that they would be receiving an invitation to participate in the near future and that their own GP was involved; in general, it also provided brief information about the trial and bowel cancer screening. They then received a standard invitation letter from the NHS national screening service in October 2009. This letter explained the rationale for bowel screening and why they have been invited; an evidence-based information booklet was also included with the letter.

We included men or women registered with a GP's practice in the south of England and who would be sent an invitation to bowel cancer screening in October 2009. We excluded those who specifically requested to be withdrawn from the programme or who were currently ineligible for invitation (i.e., current bowel cancer patients, people currently in bowel cancer surveillance programmes and so on). The age range for inclusion in the trial was 60–75 years old. The programme normally invites people aged from 60 to 69 years to screening. However, two regions were involved in piloting the age extension to the programme, which meant participants from two GP's practices aged 70–75 years were also included.

Of the people invited for screening, only one from each household was included in the trial. The number eligible participants in each GP's practice ranged from 48 to 115 (median=59), and the total number of people randomised for the trial was 1288. These were randomised to four groups: (1) GP's endorsement letter, (2) enhanced procedural instruction leaflet; (3) GP's letter plus leaflet; and (4) no additional intervention (usual care). The GP's letter and leaflet were sent out with the occult blood test kits after a week of sending the invitation letter.

### Randomisation procedure

**Figure 1:**
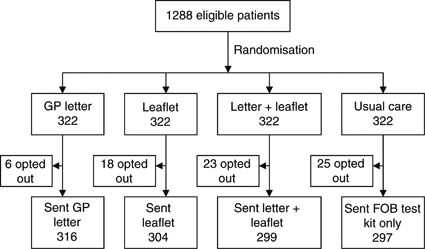
Trial flowchart.

We randomised using a block randomisation using the ‘ralloc’ command in STATA Version 10 (Timberlake, UK); allocation was concealed to the researchers, practitioners and screening hub staff responsible for recording test-kit returns. There were 644 people randomised to the two factorial groups (322 people allocated to each of the four intervention groups, see Figure 1). As the standard invitation letter allows people to opt not to be sent the test kit, not all those randomised received the intervention.

### Sample size

The sample size was based on detecting a difference in main effects between the two factorial groups (e.g., GP's letter *vs* no GP's letter, or leaflet *vs* no leaflet), not a difference between the four intervention groups (McAlister *et al*, 2003; Montgomery *et al*, 2003). The planned sample size of 387 participants per factorial group provided 80% power (*α*=0.05) to detect an absolute difference between the two factorial interventions of 10% the interventions were thought to function independently, and the study was not powered to detect interaction. It was not thought feasible to conduct a larger study with the resources available, although a difference of ∼10% was recognised to be of potential public health importance, particularly given the low cost (and hence potential for cost-effectiveness) of the interventions.

### Main outcome and statistical method

The primary outcome of the trial was verified at return of the test kit to the screening hub within 20 weeks of being invited to screening. We performed data analyses using STATA version 10. The primary analysis was conducted on an intention-to-treat basis; a per-protocol analysis was also performed for people who were actually sent a test kit and hence were subject to the intervention. The statistical significance of the main intervention effects was assessed using a multiple, random effects logistic regression model, adjusting for the other intervention and five covariates (age, gender, GP's practice, previous invitation, inclusion or not of a GP's signature) (Green *et al*, 2002; McAlister *et al*, 2003; Montgomery *et al*, 2003). As the outcome rate was >10%, the adjusted odds ratios from the regression model were used to estimate rate ratios by Zhang's method (Zhang and Kai, 1998).

## Results

The characteristics of those participating in the trial, including whether or not they received the intervention and a comparison of the factorial groups, are shown in Table 1. There were no statistically significant differences for gender, age group or previous invitation to screening between the four intervention groups. People randomised to the ‘GP's letter and leaflet’ group (*P*=0.005) and the ‘GP's letter’ factorial group (*P*=0.004) were more likely to receive the FOBT kit than other participants.

Figure 1 shows that 322 people were allocated to each of the four intervention groups, but 72 people receiving the screening invitation exercised the option of not to receive the kit (and therefore did not receive the intervention).

Table 2 shows the response rate in each of the three intervention groups individually and the comparison between the factorial groups. Both the GP's endorsement letter and the enhanced procedural information leaflet, each increased participation above usual care by about 6% – the GP's endorsement letter from 52.3 to 58.1% (absolute difference 5.8%, 95% CI: 4.1–7.8%); the leaflet from 52.2% to 58.2% (absolute difference 6.0%, 95% CI: 4.3–8.1%). The return rate in people receiving both interventions was 61.2% (absolute difference from usual care 11.8%), suggesting the effect of both interventions is additive (i.e., the absolute difference of GP's letter 5.6% and leaflet 5.9%, together is 11.5%). The absolute difference of ∼10% in the return rate for people receiving both interventions suggests a relative improvement of around 20% on current rates of participation in the English Bowel Cancer Screening Programme. The proportion of people participating in screening was higher for those receiving a signed GP's endorsement letter (64.9%) in comparison with people who received the non-signed (on behalf of the practice) endorsement letter (54.1%); an absolute difference in screening participation of almost 11%.

This additive effect was confirmed by the random effects logistic regression model (Table 3), which shows there is no suggestion of a significant interaction between the two interventions (odds ratio 1.02, *P*=0.979).

In the intention-to-treat analysis, the logistic regression model indicated that there was no significant effect on the kit return-rates by age (*P*=0.77), GP's practice (*P*=0.66), gender (*P*=0.20), GP's signature (*P*=0.16) or previous invitation to screening (*P*=0.56). Both the ‘Leaflet’ (*P*=0.029) and ‘GP's letter’ (*P*=0.038) was significantly associated with an increase in participation. In the per-protocol analysis, the insertion of an electronic GP's signature on the endorsement letter was associated with increased participation (*P*=0.039), and again there was no significant association with other covariates.

The adjusted odds ratios associated with each intervention are also reported in Table 3. On the basis of intention-to-treat, the adjusted rate ratios calculated from these odds ratios were 1.11 for the GP's letter (*P*=0.038) and 1.12 for the enhanced leaflet (*P*=0.029), and slightly lower (1.06 and 1.08, respectively) when the analysis was restricted to those who received the intervention (per-protocol analysis); reflecting the fact that the return rate in the no-treatment groups (61% and 62%, respectively) is higher when patients who opt out of screening are excluded from the denominator (number of people receiving the FOBT kit, rather than total number invited).

## Discussion

### Main findings

A letter of endorsement from the GP and a how-to-do-it procedural leaflet sent with the FOBT, each appears able to achieve a small, but important, increase in participation in the National Bowel Cancer Screening Programme. The effects appear to act independently of each other and would be additive in practice. The non-randomised comparison of the effect of a GP's signature also suggests that it is better if the endorsement letter is signed by the patient's own GP rather than by the more impersonal ‘on behalf of the practice’.

### Relation to previous findings

The findings are consistent with previous research from other countries, which have demonstrated that personalised invitations improve screening test-return rates (Brawarsky *et al*, 2004; Segnan *et al*, 2005; Cole *et al*, 2007; Senore *et al*, 2010; Zajac *et al*, 2010) and emphasise the important role that invitation materials can have on participation in screening (Steele *et al*, 2010). Furthermore, the per-protocol analysis revealed this effect is mediated by the inclusion of a GP's signature on the endorsement letter. Similar to previous research (Miller *et al*, 2005; Stokamer *et al*, 2005), the intervention would seem to enhance participants’ perceived self-efficacy to complete the FOBT kit.

The additive effect observed in the trial may reflect the influence that each separate intervention had on two distinct groups of potential non-participants. For example, Senore *et al* (2010) found those with a higher education, generally based their decision to participate in screening after reading the information materials, whereas people with a lower education tended not to read the information materials and rely on their GP's advice instead. Alternatively, people with limited health literacy (and associated lower education status) report more barriers to complete FOBT testing (Peterson *et al*, 2007). The brief enhanced procedural leaflet may have been sufficient for people with limited health literacy to address these barriers and engage in bowel cancer screening. It is, therefore, plausible that the GP's endorsement letter was influential in those who preferred receiving health advice from their GP, whereas the procedural leaflet helped people overcome perceived barriers to complete the FOBT kits. Further research directly evaluating process variables such as health literacy, the effects of gain-framing *vs* loss-framing, and the importance of GP's recommendations for bowel cancer screening are required.

### Limitations

The main design weakness was the potential for patients to opt-out of the trial after randomisation, but before the intervention was delivered. This led to a slight imbalance in the intervention groups, but there is no suggestion that this had an important effect on the results. Similarly, although the power of the study to demonstrate interaction was limited, there was no suggestion of important interaction between the two interventions. The lack of an individual GP effect on test-return in the regression analyses suggests the findings are likely to be generalisable across practices. The relatively low GP participation rate and the inability for a number of GP practices to provide an electronic signature may have implications for the widespread adoption of the endorsement letter for bowel cancer screening.

A further concern was the possibility of a ‘priming effect’ as participants were told about the trial and informed they would receive an invitation to screening 6 weeks in advance. In one previous study, participation in screening was increased when people received an advanced notification letter before their invitation (Cole *et al*, 2007). However, as the usual care group in our trial did not participate in screening at a rate much above the national average for participation in the National Programme, priming did not appear to have an important effect.

### Implications and conclusion

The results emphasise that a minor amendment to the way screening is conducted can have important effects on uptake rates. Adding a GP's letter and a more explicit instruction leaflet appears able to increase participation by at ∼10% (potentially providing a 20% relative improvement in the current participation rate). However, as less than half the GP practices recruited to this trial provided an electronic signature to the screening hub on request, this suggests there is a lack of GP engagement with the programme. Including bowel cancer screening uptake as a QOF indicator may provide the necessary incentive to remedy this lack of engagement.

Given the low cost of including a GP's endorsement letter and more explicit how-to-do-it leaflet, it is very likely that the two interventions would be cost-effective. Sending the initial invitation from GPs might also reduce the level of initial opt-out, but the potential gain in participation would have to be weighed against the increased cost and administrative complexity.

## Figures and Tables

**Table 1:** Participant characteristics for the four intervention groups and two factorial trial groups

**Intervention groups**	**Letter and leaflet**	**GP letter only**	**Leaflet only**	**Usual care**	**Total**	***P*-value**
*Gender*						
Male	153	153	154	151	611	0.996
Female	169	169	168	171	677	
						
*Age group (in years)*						
60–64	187	189	183	189	748	0.864
65–69	125	119	131	123	498	
70+	10	14	8	10	42	
						
*Previous invite*						
Yes	44	38	41	47	180	0.508
No	278	284	271	275	1108	
						
*Sent FOBT*						
Yes	316	304	299	297	1216	0.005
No	6	18	23	25	72	
						
*GP signature*						
Yes	123	116	—	—	239	0.954
No	199	206	—	—	405	
Total	322	322	322	322	1288	
						
**Factorial trial groups**	**Letter**	**No letter**	***P*-value**	**Leaflet**	**No leaflet**	***P*-value**
*Gender*						
Male	306	305	0.956	307	304	0.867
Female	338	339		337	340	
						
*Age group (in years)*						
60–64	376	372	0.583	370	378	0.513
65–69	244	254		256	242	
70+	24	18		18	24	
						
*Previous invite*						
Yes	82	98	0.199	95	85	0.422
No	562	546		549	559	
						
*Sent FOBT*						
Yes	620	596	0.004	615	601	0.089
No	24	48		29	43	
Total	644	644		644	644	

Abbreviations: GP=general practitioner; FOBT=faecal occult blood test.

Letter sent with GP's signature rather than signed ‘on behalf of the practice’.

**Table 2:** Number of people returning faecal occult blood test kits within 20 weeks according to individual intervention group, factorial group and whether or not the endorsement letter was signed by the patient's general practitioner

	**No.**	**Total**	**Percentage**	**95% CI**	**Difference (in %)**	**95% CI**
*Individual groups*						
Letter+leaflet	197	322	61.2	56–67	11.8	8.5–16
GP letter only	177	322	55.0	49–61	5.6	3.3–8.7
Leaflet only	178	322	55.3	50–61	5.9	3.6–9.1
Usual care	159	322	49.4	44–55	—	—
						
*Factorial groups*						
Letter	374	644	58.1	54–62	5.8	4.1–7.8
No letter	337	—	52.3	48–56	—	—
Leaflet	375	644	58.2	54–62	6.0	4.3–8.1
No leaflet	336	—	52.2	48–56	—	—
						
*GP letter*						
Letter signed^a^	155	239	64.9	58–71	10.8	8.6–14
Letter not signed	219	405	54.1	49–59	—	—

Abbreviations: CI=confidence interval; GP=general practitioner.

a Letter sent with GP's signature rather than signed ‘on behalf of the practice’.

**Table 3:** Likelihood of patients returning a faecal occult blood test kit within 20 weeks: results of the logistic regression analysis

**Participation (ITT analysis)**	**Odds ratio**	**95% CI**	**Rate ratio^a^**	***P*-value**
*Intervention main effects* b ^,^ c				
Letter	1.26	1.01–1.58	1.11	0.038
Leaflet	1.28	1.03–1.59	1.12	0.029
				
*Interaction* b				
Letter and leaflet	1.02	0.66–1.58	—	0.979
				
**Participation (sent FOBT kit)**	**Odds ratio**	**95% CI**	**Rate ratio^a^**	***P*-value**
*Intervention main effects* b ^,^ c				
Letter	1.17	0.93–1.47	1.06	0.186
Leaflet	1.23	0.98–1.56	1.08	0.073
GP signature	1.29	1.01–1.63	1.11	0.039
				
*Interaction* b				
Letter and leaflet	0.91	0.58–1.44	—	0.697

Abbreviations: CI=confidence interval; FOBT=faecal occult blood test; ITT=intention to treat.

a Estimated as OR/(1−*P*)+(OR × *P*) where OR is the odds ratio and *P* is the proportion of kits returned in those not receiving the intervention.

b The reference category for each main effect was those not receiving the intervention.

c Obtained for model without interaction; each intervention adjusted for the other.

## APPENDIX A

### Why are bowel motions tested?

The NHS Bowel Cancer Screening programme sends everyone aged 60–69 a faecal occult blood test (called the kit) every two years. ‘Occult’ means ‘hidden’ and the kit can detect traces of blood that you normally cannot see in your bowel motions. Blood in your bowel motions can be caused by many things – it *does not* always mean that you have bowel cancer.

You will need to take a sample of your bowel motion on three separate visits to the toilet. If you keep everything you need within reach of the toilet, it will be easier to do the test.

### It will take too much time

Collecting a sample of your bowel motion should only take a minute or two of your time. Make sure you have everything you need for the sample collection. If you have the kit within easy reach when you are sitting on the toilet, this should make it even quicker. As well as your kit, you will need a pen and whatever you choose to use to collect your sample.

### It will be too messy

There are a number of easy ways to make sure that collecting your sample is not too messy or unpleasant. Take a moment to think about which way would suit you best from the following ways:

- *Fold several pieces of toilet paper* over your hand and catch part of your bowel motion before it goes in the toilet water.
- A *plastic or rubber glove* with the folded toilet paper (you can get these from a chemist or supermarket).
- Wrap a *small plastic bag* around your hand (you can also use this to dispose of the cardboard sticks in an outside bin).
- Use a *small plastic container* that you can firmly hold or safely rest in the toilet bowl. A clean plastic take-away container or something similar is fine. Covering the container with several pieces of toilet paper will make it easier to dispose of the bowel motion and clean the container.

*For people with loose bowel motions or diarrhoea:* You can still do the test. Having loose bowel motions will not affect the test result. It may be easier to use a container to collect your samples if you have loose bowel motions.

*For people who have irregular bowel movements:* Constipation is common; it affects about 1 in 8 people. It may take a little longer to collect your three samples, but you can still do the test. After taking your first sample, you still have 14 days to take two more samples and return the kit.

### Storing your sample

It is a good idea to keep your kit in a handy place near your toilet. You will need to keep your kit in a handy place near your toilet. Please remember to keep the kit away from direct sunlight or heat. Although the kit is unlikely to smell, you can:

- put the kit in an envelope;
- keep the kit in a container with a lid.

### Returning your kit

You have 14 days after taking your first sample to return the kit to the Screening Centre. If at all possible, try to post the kit earlier in the week as kits are not tested on weekends. The hygienic envelope is completely safe to send by post.

### Any questions?

If you have any questions about the kit or bowel cancer screening then you can call:

Freephone: 0800 707 60 60

All calls are answered by trained staff and are dealt with in the strictest confidence. Please do not feel embarrassed to ask for more information or advice.

### Keep an eye on symptoms

Although screening can improve your chances of detecting bowel cancer, it is very important to keep an eye out for any of the following symptoms:

- a persistent change in bowel habit, especially going to the toilet more often or diarrhoea for several weeks;
- bleeding from the back passage without any obvious reason;
- abdominal pain, especially if it is severe;
- a lump in your abdomen.

Please see your GP if you develop any of these symptoms.

## APPENDIX B

### Help with the test

**Tips and advice on how to collect, store and return your bowel cancer screening kit**

*Should i do the test?*

This leaflet is for people who:

- do not think they are at risk of bowel cancer;
- are not quite sure how to collect their three samples;
- think that it will take too much time;
- think that it will be too messy;
- suffer from constipation or loose bowel motions;
- are a bit embarrassed to ask for help.

If you see yourself as one of these people, then this leaflet will give you advice and easy-to-follow suggestions on how you can complete the NHS Bowel Cancer Screening kit.

*Returning your screening kit can:*

- reduce your risk of dying from bowel cancer;
- increase the chance of detecting a bowel polyp before it develops into bowel cancer.

Bowel cancer is the second leading cause of cancer deaths in the UK (more than breast or prostate cancer). Eight out of 10 people who are diagnosed with bowel cancer are aged over 60. Both men and women are at risk of developing bowel cancer.

## APPENDIX C

### GP Letterhead

**Supporting Bowel Cancer Screening**

Dear [*Participants Name*],

We are writing to you to express our support for the NHS Bowel Cancer Screening Programme. Bowel cancer is one of the most common forms of cancer in the UK. Most people diagnosed with bowel cancer are over 60 years old. Screening aims to detect bowel cancer at an early stage, in people with no symptoms, when treatment is more likely to be effective.

As a Practice, we strongly recommend you complete the screening kit enclosed in this pack. If you have any questions, or would like more information about screening for bowel cancer you can contact the Programme Hub on Freephone 0800 707 60 60. However, if you have any specific concerns, or are worried about bowel symptoms, and would prefer to speak to someone at this Practice in confidence, please feel free to contact us.

It is also very important that you are aware of the symptoms of bowel cancer. The most common symptoms to look out for are:

- a persistent change in bowel habit, especially going to the toilet more often or diarrhoea
- bleeding from the back passage without any obvious reason or blood in your bowel motions
- abdominal pain, especially if it is severe
- a lump in your abdomen

Most of these symptoms will not be cancer. However, if you have experienced one or more of these symptoms for more than four weeks you should contact us as soon as possible.

Yours sincerely,

## References

[bib1] Bandura A (2004) Health promotion by social cognitive means. Health Educ Behav 31: 143–164 15090118 10.1177/1090198104263660

[bib2] Berrino F, De Angelis R, Sant M, Rosso S, Bielska-Lasota M, Coebergh JW, Santaquilani M, EUROCARE Working Group (2007) Survival for eight major cancers and all cancers combined for European adults diagnosed in 1995–99: results of the EUROCARE-4 study. Lancet Oncol 8: 773–783 17714991 10.1016/S1470-2045(07)70245-0

[bib3] Brawarsky P, Brooks DR, Mucci LA, Wood PA (2004) Effect of physician recommendation and patient adherence on rates of colorectal cancer testing. Cancer Detect Prevent 28: 260–268 15350629 10.1016/j.cdp.2004.04.006

[bib4] Cancer Research UK. Bowel (colorectal) cancer – UK mortality statistics. http://info.cancerresearchuk.org/cancerstats/types/bowel/mortality/

[bib5] Cole SR, Smith A, Wilson C, Turnbull D, Esterman A, Young GP (2007) An advance notification letter increases participation in colorectal cancer screening. J Med Screen 14: 73–75 17626705 10.1258/096914107781261927

[bib6] Cole SR, Young GP, Byrne D, Guy JR, Morcom J (2002) Participation in screening for colorectal cancer based on a faecal occult blood test is improved by endorsement by the primary care practitioner. J Med Screen 9: 147–152 12518003 10.1136/jms.9.4.147

[bib7] Denis B, Ruetsch M, Strentz P, Vogel JY, Guth F, Boyaval JM, Pagnon X, Ebelin JF, Gendre I, Perrin P (2007) Short term outcomes of the first round of a pilot colorectal cancer screening programme with guaiac based faecal occult blood test. Gut 56: 1579–1584 17616542 10.1136/gut.2007.126037 PMC2095636

[bib8] Dolan NC, Ferreia MR, Davis TC, Fitzgibbon ML, Rademaker A, Liu D, Schmitt BP, Gorby N, Wolf M, Bennett CL (2004) Colorectal cancer screening knowledge, attitudes, and beliefs among veterans: does literacy make a difference? J Clin Oncol 22: 2617–2622 15226329 10.1200/JCO.2004.10.149

[bib9] Green S, Liu P, O’Sullivan J (2002) Factorial design considerations. J Clin Oncol 20: 3423–3430 10.1200/JCO.2002.03.003 12177102

[bib10] Gurusamy KS, Gluud C, Nikolova D, Davidson BR (2011) Design of surgical randomized controlled trials involving multiple interventions. J Surg Res 165: 118–127 20097373 10.1016/j.jss.2009.09.054

[bib11] Hewitson P, Glasziou P, Irwig L, Towler B, Watson E (2007) Screening for colorectal cancer using the faecal occult blood test: an update. Cochrane Database Syst Rev (1): CD001216 17253456 10.1002/14651858.CD001216.pub2 PMC6769059

[bib12] Ling BS, Schoen RE, Trauth JM, Wahed AS, Eury T, Simak D, Solano FX, Weissfeld JL (2009) Physicians encouraging colorectal screening: a randomized controlled trial of enhanced office and patient management on compliance with colorectal cancer screening. Arch Intern Med 169: 47–55 19139323 10.1001/archinternmed.2008.519

[bib13] McAlister FA, Straus SE, Sackett DL, Altman DL (2003) Analysis and reporting of factorial trials: a systematic review. JAMA 289: 2545–2553 12759326 10.1001/jama.289.19.2545

[bib14] Miller DP, Kimberly JR, Case LD, Wofford JL (2005) Using a computer to teach patients about fecal occult blood screening: a randomized trial. J Gen Intern Med 20: 984–988 16307621 10.1111/j.1525-1497.2005.0081.x PMC1490260

[bib15] Montgomery AA, Peters TJ, Little P (2003) Design, analysis and presentation of factorial randomised controlled trials. BMC Med Res Methodol 3: 26 14633287 10.1186/1471-2288-3-26 PMC305359

[bib16] National Cancer Intelligence Network (2009) Colorectal cancer survival by stage. http://library.ncin.org.uk/docs/090623-NCIN-colorectal_survival-databriefing.pdf

[bib17] Parkin DM, Tappenden P, Olsen AH, Patnick J, Sasieni P (2008) Predicting the impact of the screening programme for colorectal cancer in the UK. J Med Screen 15: 163–174 19106256 10.1258/jms.2008.008024

[bib18] Peterson NB, Dwyer KA, Mulvaney SA, Dietrich MS, Rothman RL (2007) The influence of health literacy on colorectal cancer screening knowledge, beliefs and behavior. J Nat Med Assoc 99: 1105–1112 PMC2574401 17987913

[bib19] Rothman AJ, Bartels RD, Wlaschin J, Salovey P (2006) The strategic use of gain- and loss-framed messages to promote health behavior: how theory can inform practice. J Comm 56: 202–220

[bib20] Rothman AJ, Kiviniemi MT (1999) Treating people with information: an analysis of review of approaches to communication health risk information. J Natl Cancer Int Monogr 25: 44–51 10.1093/oxfordjournals.jncimonographs.a024207 10854457

[bib21] Seeff LC, Nadel MR, Klabunde CN, Thompson T, Shapiro JA, Vernon SW, Coates RJ (2004) Patterns and predictors of colorectal cancer test use in the adult U.S. population. Cancer 100: 2093–2103 15139050 10.1002/cncr.20276

[bib22] Segnan N, Senore C, Andreoni B, Arrigoni A, Bisanti L, Cardelli A, Castiglione G, Crosta C, DiPlacido R, Ferrari A, Ferraris R, Ferrero F, Fracchia M, Gasperoni S, Malfitana G, Recchia S, Risio M, Rizzetto M, Saracco G, Spandre M, Turco D, Zappa M, SCORE2 Working Group (2005) Randomized trial of different screening strategies for colorectal cancer: patient response and detection rates. J Natl Cancer Int Monogr 97: 347–357 10.1093/jnci/dji050 15741571

[bib23] Senore C, Armaroli P, Silvani M, Andreoni B, Bisanti L, Marai L, Castiglinoe G, Grazzini G, Taddei S, Gasperoni S, Giuliani O, Malfitana G, Marutti A, Genta G, Segnan N (2010) Comparing different strategies for colorectal cancer screening in Italy: predictors of patients’ participation. Am J Gastroenterol 105: 188–198 19826409 10.1038/ajg.2009.583

[bib24] Subramanian S, Klosterman M, Amonkar MM, Hunt TL (2004) Adherence with colorectal cancer screening guidelines: a review. Prev Med 38: 536–550 15066356 10.1016/j.ypmed.2003.12.011

[bib25] Steele RJC, Kostourou I, McClements P, Watling C, Libby G, Weller D, Brewster DH, Black R, Carey FA, Fraser C (2010) Effect of repeated invitations on uptake of colorectal cancer screening using faecal occult blood testing: analysis of prevalence and incidence screening. BMJ 341: c5531 20980376 10.1136/bmj.c5531 PMC2965320

[bib26] Stokamer CL, Tenner CT, Chaudhuri J, Vazquez E, Bini EJ (2005) Randomized controlled trial of the impact of intensive patient education on compliance with fecal occult blood testing. J Gen Intern Med 20: 278–282 15836533 10.1111/j.1525-1497.2005.40023.x PMC1490069

[bib27] von Wagner C, Good A, Wright D, Rachet B, Obichere A, Bloom S, Wardle J (2009) Inequalities in colorectal cancer screening participation in the first round of the national screening programme in England. Br J Cancer 101: S60–S63 19956165 10.1038/sj.bjc.6605392 PMC2790701

[bib28] Weller D, Moss S, Butler P, Campbell C, Coleman D, Melia J, Robertson R (2006) English Pilot of Bowel Cancer Screening: an evaluation of the second round. http://www.cancerscreening.nhs.uk/bowel/pilot-2nd-round-evaluation.pdf 10.1038/sj.bjc.6604089 PMC2360273 18026197

[bib29] Woodrow C, Watson E, Rozmovits L, Parker R, Austoker J (2008) Public perceptions of communicating information about bowel cancer screening. Health Expect 11: 16–25 18275399 10.1111/j.1369-7625.2007.00474.x PMC5060422

[bib30] Zajac IT, Whibley AH, Cole SR, Byrne D, Guy J, Morcom J, Young GP (2010) Endorsement by the primary care practitioner consistently improves participation in screening for colorectal cancer: a longitudinal analysis. J Med Screen 17: 19–24 20356941 10.1258/jms.2010.009101

[bib31] Zhang J, Kai FY (1998) What's the relative risk? A method of correcting the odds ratio in cohort studies of common outcomes. JAMA 280: 1690–1691 9832001 10.1001/jama.280.19.1690

